# Not Failure, but Low Aim Is Crime

**Published:** 1897-05

**Authors:** J. G. W. Werner

**Affiliations:** Boston, Mass.


### "NOT FAILURE, BUT LOW AIM IS CRIME."[1]

—LOWELL.

BY J. G. W. WERNER, D.M.D., BOSTON, MASS.

WHEN at the close of our last meeting a member of the Executive Committee asked me if I would give a paper, or a talk, this evening, I replied, "No;" but, upon reconsideration, I could not see why I, who had so often been benefited by the words of others, should not contribute my mite as an active Fellow of the Academy.

If in originality and intrinsic value the few rambling remarks that I shall present fall short, I must ask your kind forbearance. My aim will be to bring out discussion on some of the practical every-day points of our profession, the consideration of which may be of mutual benefit.

What are some of the most essential qualities of a successful dental practitioner?

First, as stated in my prefix, a quotation from Lowell, all of us should *aim high*, for a low aim in our profession is more criminal than an *honest failure.* Conscientiousness should predominate, direct, and guide us in all our work.

Second, moral purity and integrity of character are necessary if we would win the confidence of our patients. At this critical period of psychological study and research, when from infancy and childhood we are subjected to such searching scrutiny, is it any wonder that we human beings become so sensitive that a moral

---

[1] Read before the American Academy of Dental Science, Boston, December 2, 1896.

taint of any kind is quickly felt or detected at a glance, and an instantaneous aversion or repugnance is the consequence?

Who has not experienced this feeling on coming in contact with certain persons, though they be comparative strangers, and the meeting only casual? How much more keenly must such repugnance or aversion be intensified in the close personal relation that necessarily exists between the operator and his patient, if he is not morally pure.

Then, as, so often and so aptly quoted, "cleanliness is next to godliness," the most scrupulous care and attention must be given to personal neatness in every detail, especially in regard to the hands, nails, and breath.

A simple lotion of rose-water, glycerin, and alcohol is an agreeable application to use each time after washing the hands. The operating-coat should be suitable in texture and color and always neat and clean.

In the office and the operating-room an atmosphere of immaculate neatness and thorough ventilation should prevail. It is indispensable that no odor of medicaments be detected either in the apartments or about the person of the operator. Nothing but the cleanest napery and the most thoroughly cleansed, sterilized, and polished instruments should be used, especial care always being given to the mouth-mirror. The most careful dentists insist upon individual mouth-mirrors.

As few as possible of the necessary instruments and appliances should be exposed to view, thus avoiding undue suggestion of the operation to the patient. With the constant multiplicity of equipments there is also a danger of the operating-room having the appearance of a machine-shop.

One of the most helpful and essential factors of a well-appointed office and operating-room—one that greatly facilitates the operations; that attends to the appointments and cares for the instruments, and who gives tone to the whole place—is the neat, refined, and well-trained lady assistant. In my estimation, she is an indispensable adjunct in our work.

The third essential is professional skill and ability. Nothing is of greater value and importance to the operator in this direction than the acquiring and possession of a gentle yet firm and sure touch. For what can weary and excite a patient more than the uncertain or unskilled handling of sharp instruments, where a sudden slip might lacerate the pulp or inflict a wound upon the surrounding tissues?

The awkward, noisy, or unskilful manipulation of the instruments, either by the operator or his assistant, is also a great annoyance, and produces an undesirable effect in the mind of the patient.

Much valuable time may be wasted by an unsystematic arrangement of the necessary instruments for daily use,—or from an unfamiliarity with and an unskilled use of the same,—and also by an indecision in quickly selecting the one best suited for a particular case. A good rule is to have a few carefully selected instruments, and to learn to use them well.

Fourth, tact, in my opinion, is an important factor in meeting our patients, and in successfully allaying any fear or apprehension that naturally arises in the mind of the one who is about to undergo either a surgical operation or a medical treatment. A quiet, reassuring demeanor, and a few kindly sympathetic words help greatly to accomplish this object; while nervous movements, brusque manners, harsh or careless expressions tend—especially with women and children—to create distrust and antagonism, and will repel the patient.

Another important but delicate point to emphasize is the need of our patients to understand the necessity of constant care of the mouth. A thorough brushing and massaging of the gums as well as the teeth several times daily, and an occasional scraping of the tongue are invaluable. A neglect of such care, I believe, is at the foundation of the so prevalent pyorrhœa alveolaris.

Every operator in treating decay in approximate surfaces should endeavor to fully restore contour, thereby greatly promoting the comfort of his patient and the general healthfulness of the mouth.

For the normal arrangement of the teeth is a continuous, uninterrupted grinding surface, and spaces between approximate surfaces are unclean, annoying, and detrimental.

I might suggest other subjects for our consideration, such as the treatment of root-canals, the importance and various methods of separating the teeth before operating upon them, the preference for certain filling-materials under certain conditions, etc., all of which, when rightly understood and conscientiously practised, would help us to attain that success at which we all aim; but I will speak of only one more,—namely, the great importance of good health to the operator.

Sir Benjamin Richardson, M.D., of England, thinks that the normal period of human life is about one hundred and ten years, and that seven out of ten people could live that long if they lived in the right way. They should cultivate a spirit of serene cheerfulness under all circumstances, and should learn to like physical exercise in a scientific way. "No man, he says, need be particularly abstemious in regard to any article of food, for the secret of long life does not lie there. A happy disposition, plenty of sleep, a temperate gratification of all the natural appetites, and the right kind of physical exercise will insure longevity to most people." Unfortunately for his excellent theory, this celebrated physician and author of hygienic works died from apoplexy at the age of only sixty-eight years.

Practising dentistry, like most in-door occupations, has its detrimental effects; it tends towards short life instead of longevity; and it becomes our duty to counteract the injurious effects that our calling produces. In a general way, we may say that dentists look pale; have a tendency towards nervousness; are more or less dyspeptic; have an enfeebled circulation, heart-action, and respiratory function; acquire a contracted chest, and are short-breathed.

When we take into consideration our contracted position at the chair; the inhaling into our lungs, more or less, of the exhalations from the patient, such exhalations containing one of the most poisonous known alkaloids or ptomaines in existence; as well as the general nervous, strained condition that we are in while doing minute, delicate work, and all the time trying not to hurt; or, if we do inflict pain, to have a sympathetic perception of the same; it is not strange that all these should produce an enfeebled condition of health. When we ponder this fact, we must see at a glance the desirability, yes, necessity, of taking regular out-of-door exercise.

For years I have made it a point to work in a gymnasium; to keep an erect posture; to expand the chest by practising deep and full respiration; and have endeavored to be out-of-doors at least two hours out of the twenty-four, either walking, riding horseback, or bicycling.

From years of personal experience I know the great benefit and tonic effect of regular daily cold water bathing. Nothing rests me more than to get into a profuse perspiration from physical exercise and to take a quick cold bath afterwards.

If we wish to keep in good health we must eliminate from the system the used-up, broken-down, and effete matter; and we must not forget that through the skin, with its miles of eliminating and perspiratory ducts, is one of the best ways to accomplish this object.

As professional men, we cannot place too high an estimate upon the great importance of unimpaired health. Good health so often means pure morals, cheerfulness, helpfulness, wholesomeness in every direction. Though I have spoken of good health last, I believe it is of almost the first importance, and in recapitulation, I might say that some of the important essentials of a successful practitioner are first, health; second, tact; third, professional skill, conscientiousness, and ability; fourth, integrity and moral and personal purity; and fifth, a high aim.

